# Irregular Migrant Workers and Health: A Qualitative Study of Health Status and Access to Healthcare of the Filipino Domestic Workers in Mainland China

**DOI:** 10.3390/healthcare10071204

**Published:** 2022-06-27

**Authors:** Hui Chen, Quan Gao, Brenda S. A. Yeoh, Yungang Liu

**Affiliations:** 1 School of Geography and Planning, Sun Yat-Sen University, Guangzhou 510006, China; chenhuigeo@hotmail.com (H.C.); gaoq59@sysu.edu.cn (Q.G.); 2 Department of Geography, National University of Singapore, Singapore 117570, Singapore; geoysa@nus.edu.sg; 3 School of Geography, South China Normal University, Guangzhou 510631, China

**Keywords:** healthcare access, working condition, qualitative study, irregular migrant workers

## Abstract

In public health research, the health issues of irregular and vulnerable migrant populations remain under-explored. In particular, while mainland China has become a new and popular job-seeking destination for Filipino domestic workers (FDWs), the health status of FDWs and their access to healthcare have been invisible to public and academic concerns. This paper fills this lacuna by conducting a qualitative study that investigates FDWs’ self-reported health status and their healthcare-seeking behaviors. The results show that: (1) respondents do not report significant abusive and exploitative experience because the scarcity of FDWs in China in relation to the high demand enables them a certain degree of agency in labour market; (2) while FDWs do report some health problems, they tend to resort to self-medication and food-healing; (3) the main factors influencing health-seeking behavior include the fear of deportation, language gaps, the lack of knowledge of the local healthcare system and dependence on co-ethnic networks which serves as a double-edged sword; (4) these factors also lead to hesitation in health-seeking choice between public and private hospitals, which sometimes result in delayed treatment. This paper contributes to revealing the health conditions of FDWs in mainland China and calls for more inclusive health policy to enroll foreign domestic workers into the local health system in China.

## 1. Introduction

In 2021, there were 169 million international migrant workers, among which 70.1 million were female [1]. The World Bank Organization reports that female international migrant workers mainly engage in service-oriented sectors such as domestic work [2]. Compared to other international migrant workers, female foreign domestic workers are more likely to experience health issues [3]. However, research regarding labor migrants largely focuses on the labour market, economic inequality, and social-cultural integration in relation to migrants, with relatively fewer studies delving into their health conditions and especially, their access to healthcare [4]. This is especially so for female migrant domestic workers who are often socially vulnerable. Their health conditions are largely neglected by sending and host countries as well as migration agents [5]. The neglected health of female domestic workers is primarily ascribed to their temporary status as well as their working environments in private homes, which are isolated from the public eye and community support.

Existing studies have shown that migrant domestic workers often face a number of health issues (e.g., physical and mental health problems, infectious diseases, and sexual abuse) [3,6]. The problems are tied to house and care work, such as long hours, fatigue, repetitive work, and intense interpersonal relations, which also place them in situations liable to physical and emotional strain [7,8]. First, working conditions are known to exert a significant influence on population health [9], especially on the health of migrant domestic workers [10]. Given that domestic workers’ rhythms of daily life largely overlap with and are embedded within their workplaces, working conditions therefore become an integral part of their overall well-being. For example, physical health issues related to work such as back and arthralgia pains and musculoskeletal strains are common among foreign domestic workers [11]. Mental health issues related to work include psychotic, neurotic, and mood disorders [3]. Working in private households therefore exposes domestic workers to significant workplace risks, such as abuse, exploitation, health issues, and sexual harassment [12,13]. 

Second, immigrant regulation policies also contribute to the vulnerability of migrant domestic workers. It further reinforces the uneven employer-employee relationship [14], and eventually indirectly thwarts migrant domestic workers’ access to local healthcare system [5,15]. In most areas where migrant domestic workers are introduced, such as the United Kingdom, North America, Canada, Singapore, Hong Kong and Macao SAR (the ‘Special Administrative Region’) of China, migrant domestic workers’ legal status is often bound with their employment relationship with employers, which puts them into a situation where they unwillingly accept the unfair treatment at workplaces as a trade-off for stable employment. For example, in Hong Kong SAR of China, foreign domestic workers can only stay no more than two weeks after the termination of a contract if they could not find a new employer or renew the contract and get an approved visa (https://www.immd.gov.hk/eng/faq/foreign-domestic-helpers.html#termination accessed on 8 June 2022). This policy is also called “tied visas” in the United Kingdom [16]. Strict regulation and inadequate liable protection therefore trap foreign domestic workers within poor and unsafe working conditions, which may result in health issues [17]. However, due to the fear of being deported due to job loss or their irregular migrant status, many domestic workers are forced to endure poor working conditions, such as working overtime, added workload, low wages, and limited emotional support [18]. 

While exposed to physical and mental health problems, domestic workers also face a variety of difficulties that prevent them from seeking health services in the host country. Research has shown that foreign domestic workers face inequitable access to healthcare [19]. Factors such as the lack of knowledge or information about the receiving country’s healthcare system [20], time flexibility [21], lack of insurance, and cultural and language barriers [22] impede them from seeking health services. Even though legal foreign domestic workers have insurance, some of them are unaware of their right to request that their employers provide healthcare treatment. As a result, self-treatment is both common among legal [23] and illegal migrant domestic workers [24]. Due to these social and institutional barriers, many foreign domestic workers endure illness and delayed treatment. However, existing research suggests that social networks play a pivotal role in helping migrant workers seek healthcare treatments. According to Antoniades et al. [25], social networks can promote migrant workers’ uptake of mental health services. Piocos III et al. [26] shows that by strategically using social networks, FDWs in Hong Kong SAR of China engage in various methods to collect healthcare information and resource, and participate in conversations to improve the health-related policies of their homeland. Nevertheless, the issue of health conditions and access to healthcare among migrant domestic workers deserves more concern from the governments of origin- and receiving countries, non-government organizations, and scholars.

The concerns outlined above are more likely to happen to irregular migrant domestic workers [27]. Although many foreign domestic workers obtain their work permits from the receiving country in accordance with the contract with employers, there is still a significant number of irregular migrant domestic workers who enter the host country through informal channels [9]. Due to their fear of being deported and losing their jobs, irregular domestic workers are more vulnerable [28] to violence, mental illness, and isolation [29]. The marginalized status of irregular migrant domestic workers presents numerous challenges to public health researchers.

Although there are studies exploring the health of foreign migrant domestics workers, these studies are highly concentrated on the traditional destinations of migrant domestic workers, such as European countries [9,13,30], Canada [31,32], Singapore [5], the United Kingdom [16], the United States [33], Hong Kong SAR of China [10,23,26] and Macao SAR of China [5,34,35,36]. Less attention has been paid to the working conditions and health issues of migrant domestic workers, especially irregular migrant domestic workers, in “new” destinations such as mainland China. This paper addresses this concern by exploring the working and health conditions, and access to healthcare among Filipino domestic workers (FDWs) in mainland China. 

In the past two decades, mainland China has emerged as a new and popular immigrant destination for FDWs (https://www.sixthtone.com/news/1002446/chinas-undocumented-filipino-housekeepers-eke-out-risky-living#, accessed on 20 August 2020). Since China launched the Open Door Policy in 1978, the country has experienced 40 years of rapid development. The fast economic growth and increasing integration into the global economy have enabled China to become an emerging destination for international immigration. Mainland China is attracting immigrants from different countries of origin with diverse purposes: study, business, and acquisition of high technologies [37]. Some metropolises such as Guangzhou, Shenzhen and Beijing have become popular destinations for receiving foreign domestic workers (particularly Filipinos, Vietnamese and Indonesians) [38,39,40]. The emergence of foreign domestic workers in mainland China is closely related to the growing Chinese wealthy class and their demand for high-quality home services. Research shows that China’s middle class now accounts for about 23 percent of the total population and is growing at the rate of one percent per year; the middle class, mostly concentrated in global cities such as Beijing, Shanghai and Guangzhou, has accounted for about 40 per cent of the total [41]. These middle-class families pursue a better quality of life and invest more in their children’s education by hiring foreign domestic workers who are expected to help with housework and teach their children English [42]. However, mainland China adopts a strict work visa policy for international migrant regulation. The Chinese government has not issued any nationwide policy to introduce foreign domestic workers to mainland China, with the exception of a few experimental areas such as Shanghai where foreign talents could apply to hire foreign domestic workers (https://www.hainan.gov.cn/hainan/hygq/202010/c8070fe73cbb4e968e2c4eb21ba52166.shtml, http://news.cctv.com/2017/04/06/ARTICSAsgZXN4UuTc4YdYQkx170406.shtml, accessed on 26 July 2021). In most cities in China, citizens are still not allowed to hire foreign domestic workers. In other words, China has not established a specific kind of working visa for foreign domestic workers. Therefore, most FDWs in China are irregular immigrants [36]. The restrictions of China’s migrant policy therefore leads to the shortage of foreign domestic workers, and this is not able to meet the demands of China’s wealthy families. According to the estimation by the respondents, the salary of FDWs is about US$ 1150 in mainland China, which is much higher than the salary of those who work in Singapore (US$ 218–364) (https://www.singsaver.com.sg/blog/category/guides-tips, accessed on 8 October 2020) and Hong Kong SAR of China (US$ 590) (https://www.info.gov.hk/gia/general/202109/30/P2021093000329.htm, accessed on 8 October 2020). Higher salaries attract a large number of FDWs seeking job opportunities in mainland China despite the illegal working status. Although there is no official data, according to relevant research, the number of FDWs in mainland China has increased rapidly in the past decade [38,39,40].

While they have no legal status, FDWs have a good reputation in mainland China and are at the upstream of the labor market of house-helper. As English-speaking international migrant workers, FDWs met the Chinese middle and upper-class’s cultural imagination of, and request for, internationalization. FDWs are popular among the middle and upper-class families and stand at the top of the labor market of house helpers. This is reflected in their higher salaries, as compared to the salaries of local workers (around US$ 897–971 in city H according to our fieldwork in 2020).

While there is an influx of FDWs moving to mainland China, their precarious status means that their health concerns remain neglected. It remains a critical task to capture and understand their healthcare experiences through an in-depth empirical study. This necessitates a closer examination of how the private, social, cultural, and institutional environments influence FDWs’ health conditions and their access to healthcare. Against this backdrop, we therefore pay particular attention to the health conditions, health access and lived experiences surrounding healthcare among FDWs in mainland China and compare findings obtained with those of previous studies in other areas. In doing so, we hope to provide policy references for immigration and health policymakers in mainland China to improve FDWs’ ability to access necessary public services despite their irregular migration status. 

## 2. Materials and Methods

### 2.1. Study Design

Given that the immigration status of FDWs in mainland China is irregular, they often hide themselves from the authorities and are invisible to the public eye. There is no official data available relating to the number of FDWs in mainland China, and therefore it is highly difficult to explore this topic via quantitative means. In order to best achieve our research objectives, this study employs qualitative research to explore the health condition and the experiences of healthcare treatment of the irregular FDWs in mainland China. 

As Creswell [43] and Lambert [44] explains, qualitative descriptive research creates a comprehensive summary of specific events experienced by individuals or groups in daily life. Colorafi and Evans also highlights an important justification for the use of qualitative methods in health research: “Qualitative description is especially amenable to health environments research since it provides factual responses to questions about how people feel about a particular space, what reasons they have for using features of the space, who is using particular services or functions of a space, and the factors that facilitate or hinder use.” [45] Morse contends that qualitative health research helps to understand people’s experiences of illness and identify their health needs [46]. Descriptive qualitative study enables us to understand how FDWs make sense of their working conditions and of their bodies, as well as help us to identify the barriers they encounter in accessing healthcare. 

### 2.2. Data Collection

This study was conducted between July 2020 to December 2021 in city H, a provincial capital city in mainland China. We recruited the participants through snowball sampling. This method is used in studies where samples are difficult to find [47]; for example, in marginalized groups [48,49]. A key limitation of snowballing is that the samples might be in close social networks [47,50] affecting the nature of data obtained. To avoid this limitation and to ensure that informants are attained through a variety of categories, we extended significant efforts towards reaching out to informants in different parts of the city with different working modes (live in and live out) and socio-demographic characteristics. In total, we collected data from 22 female FDWs, and this includes two key informants from whom we started the snowball sampling. The 22 informants have stayed in mainland China between 4 to 15 years. Sampling was carried out based on purposive and saturation criteria [51,52]. All informants participated in this research voluntarily. This reduces the likelihood that the informants participated in the project for financial gains and provide answers catered to the interviewer. 

To better understand the health conditions and access to healthcare among FDWs in mainland China, face-to-face semi-structured interviews were conducted, with the assistance of open-ended interview guides (Table 1 provides a summary of the interview guides). The first author conducted all interviews in English. Considering the private nature of certain immigration and health issues, in-person interviews were conducted at the respondents’ residences or boarding houses. 

The interview guide was developed based on the research objectives, the researchers’ understanding of the phenomenon via participatory observation and informed by existing literature [5,6,27,53,54]. The interview guide was first pre-tested in 2017 with the key respondents to examine its validity and comprehensibility. Subsequent amendments of the interview guide were made according to feedback from the key respondents. This process was repeated several times until no ambiguities in understanding their answers were reported and interview answers were clear. Interviews lasted from 30 min to 1.5 h. The objectives of the study were communicated to all informants, alongside the guarantee that their personal information will be strictly protected and the interview records will be kept in confidence. This reduces the likelihood that the interviewees provide misleading information or “politically correct” answers due to the fear of potential risks such as information leakage. 

### 2.3. Informants’ Demographic Profiles

The age of the 22 female FDWs in this study ranges from 27 to 56 (see Table 2). The average age of FDWs was 41.59 years old (SD = 7.74). All participants are mothers with an average of two children each (M = 2.32, SD = 0.87). On average, these Filipinos had been working in mainland China for 7.82 years (SD = 2.46), carrying out domestic work for 13 years (SD = 6.09). Four informants had only ever worked as a domestic worker in mainland China, while the remaining 18 informants have worked in at least one other country, especially the countries in the Middle East, Southeast Asia and East Asia.

### 2.4. Data Analysis

Interview data was analyzed via Braun and Clarke’s [55] framework of thematic analysis and data sorting. The first and corresponding authors developed initial codes independently through reviewing the transcripts of the first five informants. Then, the two sets of codes were merged to form a new set of codes. Researchers used the modified codes to describe the first five transcripts. This process continued until the first five transcripts were fully reviewed and the researchers reached an agreement on the coding scheme. The coding scheme was then applied to explore the remaining transcripts and would be modified in accordance with new information. This process continued until no new themes were generated from the succeeding interviews. For this study, data saturation was achieved after the 12th interview, and the remaining transcripts did not generate new codes. These remaining transcripts confirmed that all codes reached theoretical saturation [56]. 

This study follows the guidelines of the Consolidated Criteria for Reporting Qualitative research (COREQ) [57] (Table S1). When the researchers agreed on the coding scheme, all transcripts then proceeded to software-based data sorting with the help of NVivo 12 plus. All extracted quotations in the empirical sections come from the coding scheme assisted by NVivo software. 

### 2.5. Ethical Considerations

This study was approved by the Research Ethics Panel of the corresponding author’s affiliation. All respondents were informed of the purposes of the study at the beginning of the interview. All participants in this study also voluntarily accepted the interview without any financial incentive and they were asked to sign an informed consent form. All research participants were informed that they could freely withdraw from the interview at any point. All respondents were anonymized, which was especially important given the fact that they were irregular migrant workers.

## 3. Findings

This section presents respondents’ working and health conditions, their efforts to maintain health, and their lived experiences of accessing healthcare (including barriers to accessing healthcare in mainland China). Table 3 summarizes the main themes generated from the data sorting process of this study and are supported by quotations from the interviews. 

### 3.1. Working Conditions of FDWs in Mainland China

Existing studies show that female migrants and especially domestic workers are more likely to be embedded in vulnerable conditions [3,58,59]. In this study, the FDWs in mainland China we interviewed report a relatively strong agency in choosing and negotiating their working environments. The employment agency of FDWs in mainland China primarily manifests in their ability to negotiate job contents, working hours and salary with employers. Rather than being passive receivers of working conditions set by employers, they will ask the employer to increase their wages if their work exceeds the agreements they made before.

*“I have stayed with the family for nine years. Before, I told the madam that I want to quit the job. She asked me why, I told her, the basic salary is higher than what I have now. She told me, don’t go, and she raised my salary to persuade me to stay.”*

(F17, 53-year-old)

*“If I think, the job is too hard, tired, and the salary is not good enough, I will quit the job; it is easy for us to find a new job.”*

(F3, 46-year-old)

Respondents did not report any experience of being abused and exploited during work. In this study, the mean working hours of FDWs in mainland China is about 10 h per day. Although they work more than 8 h per day, they claim that their work is relatively flexible and not intense. For example, most FDWs claim that their work “is not heavy” for them:

*“I work from 8:30 am to 9 pm, 12 h, one day off every week. In the morning, I start at 6:50. From 2:00 pm to 4:00 pm is my nap time. Not too much work.”*

(F17, 53-year-old)

*“I work 8 h* *ours one day. I need to get up at 5/6 AM in the morning to prepare food for the kids. Sometimes I go to bed at 12 am at night, after the kids fall asleep. I don’t work for the whole day, when I finished the work, I could have a rest.”*

(F8, 36-year-old)

Working hours often depends on the nature of housework and child caring. Sometimes it is hard to ensure that the actual working hours fall within the working hours that the contract designates. However, FDWs in this study note that no significant abuse or exploration occurred:

*“I work from 6:30 am to 10 pm every day and have one day off every week. In fact, we can’t count how many hours for work, just until everyone is ready to go to bed in the night. In fact, there is no limit of time. I am not keeping on work all the time, could have rest during the daytime.”*

(F6, 39-year-old)

*“If I work overtime or I have to work on my rest day, my boss will pay for that. Like, if they need me to work on my rest day, they will pay for that.”*

(F8, 36-year-old)

While other research suggests that domestic workers suffer various types of mistreatment in other countries [58,60], none of the respondents reported ever experienced discrimination and sexual abuse in this study. All respondents even gave relatively positive assessments of their employers:

*“My employers are all very nice to me.”*

(F2, 36-year-old)

*“I think no abuse here, in China. The Chinese employer and the Chinese are good. They have good temper.”*

(F15, 42-year-old)

In general, there are at least three factors that contribute to FDWs’ relatively friendly or less exploitative working environments in mainland China. First, FDWs are considered to have privileged cultural and human capital characterized by their English-speaking skills and their professional identity in comparison with the local Chinese domestic workers. Therefore, FDWs are often in shortage and highly sought after in mainland China’s labor market of domestic workers. This endows them with a certain degree of agency in improving their circumstances. Second, the cultural barriers between Chinese employers and FDWs collectively produce a “respectful distance” between FDWs and the employers, which not only avoids intimate interactions but also minimizes the occurrence of abuse and exploitation. Third, as China has no work visas for FDWs that formalizes the legal relationship between employers and employees, FDWs could find a new employer easily when they are unsatisfied with their current employers. This agency in employment enables FDWs to have more say in job negotiations and therefore reduce the risk of exploitation. This is why most FDWs in this study have positive evaluated their employers. 

### 3.2. Health Conditions of FDWs in Mainland China

#### 3.2.1. Health Condition

Most respondents’ self-evaluations of their health conditions are positive even though they consciously know that they have some health problems. Their self-evaluations of health conditions are based on pre-work occupational medical checks. Except for those who had prior surgical experience in mainland China, all other informants have never had a general physical check-up in mainland China. Most of the informants have a misconception about their health condition: they usually believe that they are relatively healthy. They often confuse occupational medical check-ups (that only examine certain items) with general physical check-ups. Every time they change jobs, the new employer will ask the worker to have an occupational medical check-up in advance. This kind of medical check-up only targets specific infectious diseases such as HIV, Tuberculosis, Chronic Hepatitis B, Helicobacter pylori, and Syphilis.

*“I have medical check; it is for new job. Although it is not the whole body check, I think that is enough, I don’t have any infectious diseases.”*

(F21, 23-year-old)

*“I have medical check…Oh, it is not a whole body check. Every time I prepare for another job, I will have a medical check. Like Chronic Hepatitis B and X-ray.”*

(F18, 45-year-old)

Despite their general confidence in self-evaluation of their health conditions, most of the informants also report different degrees of physical and mental health issues. The most common physical health problems mentioned are related to osteoarticular and muscular issues such as back pain, but they also note that these physical health issues are not just related to the current work, but have been accumulated over a long time, as most informants had engaged in domestic work for many years before coming to China.

*“I am healthy. While my shoulder, back and my lower back always pain, sometimes it is hard for me to bend over.”*

(F7, 38-year-old)

*“I have hurt my back before. I often feel painful.”*

(F11, 50-year-old)

Gynecological disease and varicose veins are also common physical issues reported by many informants. In particular, varicose veins and ovarian cyst are the most common health problems. They verbalized their symptoms as follows:

*“I have ovarian cyst issue. I’ve seen the doctor, the doctor said it was a benign tumor. So, I let it there…. I didn’t have medical treatment.”*

(F13, 36-year-old)

*“I am healthy. Oh, I have varicose veins on my legs. And I always feel painful of my legs. I always wear kneecap even in summer when I stay in the air-conditioned room.”*

(F18, 45-year-old)

Although no deliberate and noticeable abuse and exploitation imposed by the employers are reported, some respondents declare that they are sometimes troubled by mental health problems that are associated with the contents of domestic work (e.g., caring of children). Taking care of and accompanying children often causes mental health issues such as stress. Some respondents describe:

*“I am healthy. But sometimes I am not feeling very good. I can’t sleep well if I change place, change bed. Maybe I am old, haha…”*

(F17, 53-year-old)

*“The kids always cry, I really feel stressful. It is really hard to endure. I am looking for a new job.”*

(F7, 38-year-old)

Moreover, their irregular immigration status also contributes to the mental health problems experienced by FDWs in mainland China. Worrying about their migration situation may even lead to certain physical health issues. Some respondents say:

*“When the check is strict, I really feel tired, my heart is not peaceful.”*

(F3, 46-year-old)

*“It is hard for me to fall asleep, even though I am tired. I am losing a lot of hair. I am worried, hair is important for women, if it kept losing, I will be looked very old.”*

(F3, 46-year-old)

#### 3.2.2. Ways of Keeping Fit

According to our investigation, food is considered to be the most important way to keep fit for FDWs. For them, food is also supposed to be a compensation for their hard work. Some informants gained weight since they came to China. The emphasis on food by FDWs may be associated with Filipino eating culture and attitude. According to Kayano et al.’s (2008) cross-cultural research on eating attitudes and body dissatisfaction, Filipino exhibit a much weaker desire for thinness in comparison with people from Western countries and East Asia [61]. Instead, for FDWs, eating is invested with meaning, related to ethnicity and occupation.

*“Eat more, we need to be strong, we need to eat more. We are doing hard work; we can’t get sick.”*

(F1, 50-year-old)

*“Eat more, keep healthy and strong. I am fatter than before. I also want to lose weight, but it doesn’t matter.”*

(F7, 38-year-old)

Food was seen not only as a way to sustain life and strengthen the body, but also as a means to strengthen ethnic and occupational identity.

*“Eat more, our work is hard, we need to be strong. Chinese people are generally thin. My boss doesn’t eat rice for dinner. But we need to be strong.”*

(F8, 36-year-old)

*“Eat more, it will help you keep healthy and strong. I know Chinese don’t like to be fat. My boss limits the amount of food she takes every day. She wants to keep slim.”*

(F5, 41-year-old)

Some informants hope to keep healthy by taking healthcare products. One respondent claims to take vitamin D and grape seed (F1, 50-year-old) while another takes a multivitamin that helps her “have a good sleep” (F3, 46-year-old).

### 3.3. Barriers to Accessing Healthcare

According to our study, the main determinants that impede the FDWs’ utilization of health service in mainland China include the fear of accessing health services due to their irregular status, language and cultural barriers, alongside limited access to healthcare information.

#### 3.3.1. Irregular Status and Fear of Deportation

Our research shows that undocumented or irregular workers exhibit a much lower incentive to seek health services. A major barrier that impedes FDWs’ access to public healthcare is their irregular migrant status, which they think will expose them to the risk of being detected and deported by the police. 

The medical care system in mainland China is organized within the National Health System (NHS), funded through the taxation of its citizens. Healthcare is delivered through a variety of channels in mainland China, including private and public hospitals, private clinics, and international hospitals. The public health facilities in mainland China are viewed to be of high quality by local citizens. Every public hospital will assign a unique healthcare identification number for each patient, and the number is bound to an ID card or passport number such that the case report of each patient could be recorded and tr acked. Although patients are required to register their address when seeking medical care from hospitals, medical data information is normally not linked to the immigration regulation system. Nevertheless, FDWs fear that they will be asked for their papers (e.g., visas [F3, 46; F6, 39]), so they often avoid visiting hospitals to keep a safe distance from China’s bureaucratic system. Some FDWs describe the following:

*“It is hard for us to go to (the) hospital: we don’t have a paper. So, it is important to keep fit.”*

(F9, 56-year-old)

*“I have been to the public hospital. While, I’m afraid of going to the public hospital. I don’t know what will happen, I have to make sure nothing will be happened, for me, safety is the first important thing. If anything happens to me, who will send money to my kids in the Philippines?”*

(F18, 45-year-old)

*“I didn’t dare go to the hospital before, I fear… they will check on you, and you need to register and show an ID. I think it is dangerous. I want to stay here; I need to work.”*

(F22, 48-year-old)

It is worth noting that a large proportion of FDWs do not hold work visas issued by the Chinese Immigration Bureau, but come to mainland China with the tourist visa. As such, their irregular migrant status places a significant psychological burden on them in seeking medical treatment and, to a certain extent, prompts them to isolate themselves from the Chinese public healthcare system.

#### 3.3.2. Language Barrier

The language barrier is another constraint FDWs face when accessing healthcare in mainland China. Although English education is becoming more popular in mainland China, many doctors and nurses are still not able to achieve barrier-free communication with patients in English. At the same time, although most hospitals are equipped with English signages, application forms and testing results are only presented in Chinese. It is hard for foreigners to communicate with doctors due to the lack of knowledge and local language skills.

*“The biggest difficulty is language. I don’t know Chinese and I can’t communicate with doctors.”*

(F5, 41-year-old)

*“I don’t understand Chinese, I can’t communicate with the doctor.”*

(F3, 46-year-old)

Even though some FDWs have stayed in mainland China for a long time, the language barrier is still hard to overcome and therefore serves as one of the most crucial factors that prevents them from seeking healthcare.

*“The last time, I went to see the doctor, I don’t understand, and can’t communicate with him. My Chinese friend helps me communicate with the doctor. “The doctor prescribed medicine for me, but that is only for about 1 weeks, I need to see the doctor for the second time. But I didn’t go to see the doctor again. It needs help.”*

(F3, 46-year-old)

*“I don’t understand Chinese, I don’t know how to do. For the first time, I am afraid there is checking of ID. Now, I know that they won’t ask for visa, only name and your passport ID. But I still don’t know how to do.”*

(F4, 33-year-old)

The quotations above indicate that the lack of social networks and support from local people exacerbates FDWs’ fears of seeking medical services from public hospitals.

#### 3.3.3. Limited Access to Healthcare Information

The lack of knowledge regarding the healthcare system is another significant barrier FDWs face when accessing healthcare. For most FDWs, ethnic social networks are the main sources of medical information that would guide them to seek medical treatment. Seeking help and information from ethnic social networks (i.e., Filipino friends) is the first step before they visit a doctor.

*“I will ask my friend about the information, they have stay here for a long time. They know which hospital we could go and how to get there, and which one is cheaper.”*

(F14, 38-year-old)

*“I learned that one of my friends successfully see a doctor in the hospital, yes, the public hospital. I asked her how she did it and if she could help me.”*

(F20, 36-year-old)

It must be noted that joining a social group is a double-edge sword for FDWs’ ability to access healthcare. On one hand, ethnic social networks can provide FDWs with information and help. On the other hand, disinformation obtained by their Filipino friends via ethnic social networks also prevented them from accessing healthcare in timely and correct ways. In many circumstances, FDWs were misguided by their ethnic friends to unlicensed clinics that may instead deteriorate their health problems.

*“Yes, I will ask Filipinas, but some of them are stupid, haha. I mean, some of them don’t know the real situation, they just believe what the other said.”*

(F1, 50-year-old)

*“My friend advised me to go to an unlicensed clinic, she gave abortion there. The doctor is a woman and could speak English. I talked with her online via WeChat. It is too expensive. I didn’t go to that unlicensed clinic.”*

(F10, 27-year-old)

*“I asked my friend about which hospital is proper. They suggested me a private clinic, and told me that the hospital my employer arranged for me is not good. I am not sure.”*

(F17, 53-year-old) [Note: the public hospital her employer arranged is a tertiary hospital *.] [*Note: In mainland China, a tertiary hospital is a comprehensive and referral, hospital with a bed capacity exceeding 500. They are normally considered as the most competent hospitals in providing medical service, medical education and scientific research.]

Bernadas and Jiang’s (2016) research on the health of FDWs in Hong Kong SAR of China suggests that employers normally serve as an important resource that FDWs would turn to [20]. Nonetheless, in our study, FDWs in mainland China are reluctant to ask for help from their employers. They offer various reasons:

*“I ask help from my Filipino friends to see how to do. I will not ask my employer, that is not good. I can’t tell her all the things.”*

(F10, 27-year-old)

*“My boss is busy, for most of the time, I don’t bother her if I can find ways.”*

(F14, 38-year-old)

*“I won’t ask my boss for a suggestion. Not every boss is concerned. I ask my friend for advice; we help each other.”*

(F6, 39-year-old)

The quotations above further illustrate our argument that the cultural barriers often create a “respectful distance” between Chinese employers and FDWs. Therefore, this social and emotional distance thwarts them from developing more intimate and in-depth reciprocal relationships. In this sense, this “respectful distance” also reduces the possibility of humanistic protection for FDWs from employers.

### 3.4. Experience of Accessing Healthcare

#### 3.4.1. Self-Treatment

Most informants expressed that they are afraid to go to public hospitals when they have a health problem. In most cases, they prefer to self-medicate by taking medication that they brought from the Philippines and Hong Kong SAR of China or bought from local pharmacies. In particular, the Philippines’ medications and traditional herbs that they brought from their homeland are more popular:

*“When I am sick, I will have a rest and take medicine first. I will ask for leave and stay at home for one or two days, and go back to work when I am better. You don’t need to go to hospital every time.”*

(F5, 41-year-old)

*“I bought some medicines online, from Hong Kong SAR of China. I prefer the Philippines’ medicine. But they are not available always, sometimes we need to buy medicines from pharmacy.”*

(F7, 43-year-old)

Many informants also choose self-treatment even after seeing the doctor in the hospital.

*“I take the Philippines’ traditional medicine. I searched from Facebook to see how to do. Some herb could buy online. I also will keep the medicine that the doctor prescribed for me. When I am not feeling well, I will ask Chinese friend to buy the same medicines for me.”*

(F19, 56-year-old)

*“The doctor prescription medicine for me, but that is only for about 1 weeks, I need to see the doctor for the second time. But I didn’t go to see the doctor again. It needs time and money. I also search online, it is not serious, and I think I could take some healthy product, and keep fit.”*

(F3, 46-year-old)

#### 3.4.2. Private Clinics and International Hospitals

According to the respondents, if they have to see a doctor, they would prefer to visit private clinics and international hospitals. In international hospitals, there are less communication barriers and lower risks of being detected by authorities, thereby making international hospitals the preferred choice of FDWs when they need to see a doctor.

*“I prefer an international hospital. I go to the international hospital. Yes, it is expensive, but I think it is safe, and the doctor could speak English. For us, keeping safe is important. I know others go to the private clinic; they are just afraid to go to the public hospital, they are afraid to be caught.”*

(F9, 56-year-old)

*“I went to international hospital. It is safe and the doctors there could speak English. I could do it by myself. You can’t ask for help from others always.”*

(F18, 45-year-old)

#### 3.4.3. Public Hospitals

While many FDWs prefer visiting private clinics, most informants would turn to public hospitals when medical treatment is sorely needed. Due to the language barrier and worries regarding their safety, they usually ask reliable Chinese locals for help and guidance. Asking for the company of local Chinese friends not only provides them with psychological security, it also helps them better understand the unfamiliar procedures in Chinese hospitals. Moreover, some FDWs tend to prioritize public hospital over private clinics since the costs of healthcare treatments are more affordable compared to that of private clinics.

*“If I have to see a doctor, I will go to public hospital; private hospital is expensive. Most of the Filipinos are afraid to go to public hospital, they didn’t try.”*

(F5, 41-year-old)

*“I know that we won’t be caught in the hospital, but I am still feared to go to hospital. I don’t understand Chinese, it is hard. I will keep the outer packaging of the medicines that the doctors prescribed for me. Next time, when I met the same health problem, I will buy the medicines from the pharmacy.”*

(F14, 38-year-old)

*“When I am predelivery, I asked my Filipino friends about where I should go. I really need help. My boyfriend doesn’t have paper either, he can’t show up at the hospital. He also tried to find ways. Finally, we checked a public hospital. The father asked a Chinese to help us.”*

(F2, 36-year-old)

#### 3.4.4. Delayed Treatment

However, the lack of general medical check-ups, the fear of being deported as well as the cultural and language barriers may sometimes collectively lead to delayed access to healthcare and thus result in critical conditions caused by delayed treatment. During our fieldwork, we unfortunately heard of four cases of delayed treatment. One FDW was in critical condition but unable to receive timely surgical treatment; she died after receiving months of conservative treatment in mainland China. The other three FDWs chose to go back to the Philippines to undertake life-saving treatments:

*“I know I have health problem, but I think it is not serious. I don’t have paper; I am afraid to go to hospital. Until it seemed to be a little serious, I talked to my friend, they helped me to go to hospital. Public hospital. The doctor asked me to be hospitalized, we don’t know how to do. At last, we beg the help of our consulate.”*

(F22, 48-year-old)

Going back to the Philippines to seek medical treatment was the preferred choice for many FDWs we interviewed. Respondent gave various reasons:

*“I told some of my friends: if you feel sick you have ill in mainland China, it is time to go home. Because it is very hard, you don’t have family here, no one can be concerned about you, your health… No one could take care of you in the hospital. All of us have our own problems, have our own families.”*

(F15, 42-year-old)

*“My Chinese friend brought me to the hospital for gynecological issues. Some of my families also have the same issues, I am worried, if the result is not good, I want to go back to the Philippines to cut off of the relevant part.”*

(F4, 33-year-old) [Note: Her medical result revealed that she was not seriously ill. Six months after her recovery, she chose to go back to the Philippines.]

*“If I am sick, I will return to the Philippines. I have my own house and families in the Philippines, I’m not afraid of anything.”*

(F13, 36-year-old)

The lack of regulations surrounding the employment relationship between employers and FDWs has resulted in ambiguous obligations for the employers. The unclear responsibilities of employers inevitably lead to a situation that FDWs cannot legally request the assistance of healthcare from employers. 

## 4. Discussion

This study explores the working and health conditions of FDWs and attempts to reveal the barriers and vulnerable experiences that irregular migrant domestic workers face in accessing healthcare in mainland China. Although mainland China is experiencing an influx of foreign domestic workers who are mostly Filipinas, FDWs are typically left invisible in the public eye due to their irregular status. It is therefore important for policymakers and the public in China to achieve a more in-depth understanding of irregular migrant domestic workers’ health conditions and their access to healthcare.

Existing research on the working conditions of migrant domestic workers has provided evidence of their vulnerabilities to workplace abuse, exploitation, discrimination and violence [5,29,62]. However, our research findings show that despite the prevalence of labor abuses among female migrant domestic workers in other countries, the 22 FDWs we interviewed did not report significant and deliberate abuse and discrimination carried out by the employers. We relate their relatively friendly working conditions to the scarcity of FDWs in mainland China, which enable them to attain a certain degree of agency in choosing and negotiating the content and intensity of work. In general, the employment agency of FDWs in mainland China stems from two factors. First, as a new immigrant destination for FDWs, mainland China has not issued specific work visa to formalize or bind the legal nexus between FDWs and employers. Therefore, FDWs can freely switch to new employers in the informal labour markets. Second, their scarcity and perceived professionalism enable them at the upstream of the labor market of house-helpers. Thus, a thought-provoking contribution that we offer in this paper is that different immigrant regulation policies may have diverse impacts on migrant domestic workers’ working conditions [63]. In mainland China, the informal labor market due to the lack of visa-based regulations has created a relatively friendly working environment for FDWs. 

In terms of health statuses, physical health, gynecological, and mental health issues are the most common health problems reported by informants in our study. According to the respondents, these health problems are more likely related to the nature of housework and care work than the working conditions in which they are situated. Moreover, although China’s healthcare system does not exclude irregular migrants, many FDWs are still reluctant to visit the public hospital due to their fears of being detected (Note: no case indicates that FDWs were detected as a result of visiting public hospitals). Respondents who had been treated in public hospitals gave positive evaluations of their experiences with healthcare and treatment. As mentioned above, the main barriers for FDWs to access healthcare are the fears of deportation related to their irregular status, language and cultural barriers, and the limited access to healthcare information regarding the local healthcare system.

Existing studies have shown that social and ethic networks play important and positive roles in the healthcare-seeking behaviors of FDWs in Hong Kong SAR of China [20]. However, apart from highlighting that FDWs rely significantly on social networks to seek healthcare and share health-related information, we have also illustrated how ethnic networks can serve as a double-edged sword that may facilitate and misguide FDWs’ health-seeking behaviors. Filipino networks primarily provide FDWs with information regarding healthcare, but this information may also direct them to unlicensed clinics that causes harm instead. 

## 5. Conclusions

Compared to local citizens, FDWs in mainland China are still a marginalized and vulnerable ethnic group. It is undeniable that giving attention to this marginal group of foreigners may provide us with opportunities to improve Chinese immigration policies and promote social equity. In particular, given the current political, socio-economic, and healthcare challenges posed by the increasing international migration inflows to mainland China, China needs to pay attention to the health conditions of these international migrants accordingly. The findings of this study show that although no significant and deliberate workplace abuses or exploitation were reported among the FDWs, access to the local healthcare system is a significant problem for FDWs. To build a more inclusive healthcare system adequately supports FDWs in mainland China, health policymakers should first eliminate or at least reduce the institutional barriers for foreigners. In doing so, the Chinese government could develop a more inclusive health policy that enables foreign domestic workers to better access local healthcare services. For example, additional measures such as offering information and knowledge relating to China’s healthcare system as well as offering relevant language support to FDWs may facilitate their willingness and ability to visit public hospital, thereby reducing the chances of delayed treatment. These measures may help (irregular) migrant workers in mainland China to attain better access to healthcare so as to improve their health conditions.

## 6. Limitations

This paper is a qualitative study that focuses on the health conditions of 22 FDWs in mainland China. It seeks to uncover the context-based lived experiences of 22 FDWs and provide an in-depth understanding of their health-seeking behaviors. In light of this, we cannot ensure that the research findings can be generalized to all FDWs in mainland China. Other limitations of the study should also be noted. First, this study adopts the snowball sampling method, which is widely used by most quantitative studies, especially in situations where samples are difficult to find. However, the inherent risk related to snowball sampling is that it relies on selecting informants within close social networks. Although we have tried to avoid method bias when recruiting informants in our fieldwork, our method may still be unable to fully address the complexity of FDWs. Second, due to the sensitivity of the irregular migrant status, we were not able to conduct large-sample research. The 22 informants may not represent the health issues of all FDWs and their experiences in accessing healthcare in mainland China. Third, some interview questions in relation to sexual harassment at the beginning of the interview may cause discomfort to informants, especially considering the fact that trust between the interviewees and interviewer had not yet been fully built. Although we eventually gained trust from the interviewees and ensured that their privacy will be strictly protected, there was still the probability that the respondents were sensitive to questions about sexual harassment. In general, our study aims to capture the phenomena and everyday experiences of FDWs in mainland China, which has not received sufficient attention. Future research should address these shortcomings by expanding the size of sampling and by conducting comparative exploration across different cities in mainland China.

## Figures and Tables

**Table 1:** Interview guide.

Content/Example Questions
**Introduction: To learn about the working conditions, access to and experiences of healthcare of irregular Filipino domestic workers in China**
**Could you tell me a bit about yourself?** Age, families, length of staying in mainland China, length of doing domestic work, salary
**Working conditions** Could you talk about your regular working day? (weekly work schedule, daily work schedule: start, finish, rest time) Have you ever been treated unfairly at work? (Experience of abuse, discrimination, sexual harassment, over work) Could you tell me about your relationship with employer?
**Health conditions** Could you make a self-assessment of your health (Current health status)? Do you have any health problems? How do you keep fit?
**Healthcare access** **Did you encounter any difficulties when you were seeking health service in China?** If yes, what difficulties did you face? **Tell me about your decision-making about the health treatment plan.** Who will you ask for help? Will you go to see a doctor? What medicines do you take? **Can you tell me about your hospitalization experience since you came to China?** What kind of hospital did you visit? Tell me how did you choose the hospital? What was the feeling when you were seeing a doctor? What difficulties did you face when you were seeing a doctor?
**Final question** Is there anything else you would like to share about your health issues and your experiences of accessing healthcare?
**Appreciation** Express appreciation to the informants for their time and help and emphasize again that we will protect their personal information.

**Table 2:** Demographic information of informants (N = 22).

Informants	Age	Number of Children	Length of Stay in China (Years)	Length of Doing Domestic Work	Duration of Interview
F1	50	1	8	15	90 min
F2	36	2	8	7	60 min
F3	46	4	9	20	90 min
F4	33	2	5	8	60 min
F5	41	2	5	5	60 min
F6	39	2	8	15	40 min
F7	38	3	15	18	60 min
F8	36	2	4	4	60 min
F9	56	3	9	20	60 min
F10	27	3	6	6	40 min
F11	50	3	8	20	30 min
F12	37	1	9	12	30 min
F13	36	2	8	11	30 min
F14	38	4	8	11	90 min
F15	42	2	9	18	60 min
F16	40	2	5	9	60 min
F17	53	3	12	25	60 min
F18	45	1	7	20	40 min
F19	56	3	8	20	30 min
F20	36	3	8	16	60 min
F21	32	2	4	6	30 min
F22	48	1	9	20	30 min

**Table 3:** Findings presented as themes and subthemes.

**Working condition**
No significant abuse, exploitation and sexual harassment
Easy to find new jobs (FDWs show strong agency in job negotiations)
The ways FDWs were being treated at workplaces
**Barriers to access to healthcare**
Fear to access public healthcare (fear to be asked for visa, fear to be tracked and deported, can’t communicate with doctors)
Language barriers (barriers to get information, can’t communicate with doctor, can’t fill out the relevant forms)
Limited access to healthcare information (over-reliance on ethnic social networks)
**Decision making**
Ethnic social networks (main way of health information-seeking)
Chinese friends
**Experience of hospitalization**
Self-treatment (have a rest, take medicine (prefer the Philippines’ medicines))
Public hospital (language barriers, fear to be tracked and deported)
International hospital (no language barrier and safe to visit, suggested by social networks and other foreign friends/employer)
Unlicensed clinic (for safe, guided by social network)

## Data Availability

The data presented in this study are available on request from the first author.

## Supplementary Materials

The following supporting information can be downloaded at: https://www.mdpi.com/article/10.3390/healthcare10071204/s1.

## References

[1] IOM (2021). World Migration Report 2022.

[2] World Bank, Migration and Development Brief 34. https://www.knomad.org/sites/default/files/2021-05/Migration%20and%20Development%20Brief%2034_1.pdf.

[3] Malhotra R., Arambepola C., Tarun S., de Silva V., Kishore J., Østbye T. (2013). Health issues of female foreign domestic workers: A systematic review of the scientific and gray literature. Int. J. Occup. Environ. Health.

[4] Liem A., Wang C., Wariyanti Y., Latkin C.A., Hall B.J. (2020). The neglected health of international migrant workers in the COVID-19 epidemic. Lancet Psychiatry.

[5] Hall B.J., Garabiles M.R., Latkin C.A. (2019). Work life, relationship, and policy determinants of health and well-being among Filipino domestic Workers in China: A qualitative study. BMC Public Health.

[6] de Diego-Cordero R., Vega-Escaño J., Tarriño-Concejero L., García-Carpintero-Muñoz M.Á. (2020). The Occupational Health of Female Immigrant Caregivers: A Qualitative Approach. Int. J. Environ. Res. Public Health.

[7] Blackett A. (2011). Introduction: Regulating Decent Work for Domestic Workers. Can. J. Women Law.

[8] Tong C.E., Sims-Gould J., Martin-Matthews A. (2016). Types and patterns of safety concerns in home care: Client and family caregiver perspectives. Int. J. Qual. Health Care.

[9] Barnay T. (2016). Health, work and working conditions: A review of the European economic literature. Eur. J. Health Econ..

[10] Chung R.Y.-N., Mak J.K.-L. (2020). Physical and Mental Health of Live-In Female Migrant Domestic Workers: A Randomly Sampled Survey in Hong Kong. Am. Behav. Sci..

[11] Ahonen E.Q., López-Jacob M.J., Vázquez M.L., Porthé V., Gil-González D., García A.M., Ruiz-Frutos C., Benach J., Benavides F.G. (2010). Invisible work, unseen hazards: The health of women immigrant household service workers in Spain. Am. J. Ind. Med..

[12] Human Rights Watch (2005). Maid to Order: Ending Abuses against Migrant Domestic Workers in Singapore.

[13] Triandafyllidou A., Triandafyllidou A. (2013). Irregular Migration and Domestic Work in Europe: Who cares. Irregular Migrant Domestic Workers in Europe.

[14] Huang S., Yeoh B.S.A. (1996). Ties that Bind: State Policy and Migrant Female Domestic Helpers in Singapore. Geoforum.

[15] Hill N.S., Dorow S., Barnetson B., Martinez J.F., Matsunaga-Turnbull J. (2019). Occupational Health and Safety for Migrant Domestic Workers in Canada: Dimensions of (Im)mobility. New Solut. J. Environ. Occup. Health Policy.

[16] Demetriou D. (2015). ‘Tied Visas’ and Inadequate Labour Protections: A formula for abuse and exploitation of migrant domestic workers in the United Kingdom. Anti-Traffick. Rev..

[17] Auerbach A., McCabe K., Whiteman E.D. (2014). A Health Impact Assessment of the Massachusetts Domestic Workers’ Bill of Rights.

[18] International Labour Organization (2013). Domestic Workers Across the World: Global and Regional Statistics and the Extent of Legal Protection.

[19] Fernandez B. (2018). Health inequities faced by Ethiopian migrant domestic workers in Lebanon. Health Place.

[20] Bernadas J.M.A.C., Jiang L.C. (2016). “Of and beyond medical consequences”: Exploring health information scanning and seeking behaviors of Filipino domestic service workers in Hong Kong. Health Care Women Int..

[21] Weng S.F., Malik A., Wongsin U., Lohmeyer F.M., Lin L.F., Atique S., Jian W.S., Gusman Y., Iqbal U. (2021). Health Service Access among Indonesian Migrant Domestic Workers in Taiwan. Int. J. Environ. Res. Public Health.

[22] Asis M.M. (2005). Preparing to Work Abroad: Filipino Migrants’ Experiences Prior to Deployment.

[23] Jabar M.A. (2021). Factors influencing health-seeking behavior among overseas Filipino workers. Int. J. Healthc. Manag..

[24] Community Health Group (2013). Health Seeking Behavior of Migrant Workers in Singapore.

[25] Antoniades J., Mazza D., Brijnath B. (2018). Agency, activation and compatriots: The influence of social networks on health-seeking behaviours among Sri Lankan migrants and Anglo-Australians with depression. Sociol. Health Illn..

[26] Piocos III C.M., Vilog R.B.T., Bernadas J.M.A.C. (2022). Interpersonal Ties and Health Care: Examining the Social Networks of Filipino Migrant Domestic Workers in Hong Kong. J. Popul. Soc. Stud. JPSS.

[27] Tsiodras S. (2016). Irregular migrants: A critical care or a public health emergency. Intensive Care Med..

[28] Office of the High Commissioner for Human Rights (2015). Behind Closed Doors: Protecting and Promoting the Human Rights of Migrant Domestic Workers in an Irregular Situation.

[29] Huang S., Yeoh B.S.A. (2007). Emotional Labour and Transnational Domestic Work: The Moving Geographies of ‘Maid Abuse’ in Singapore. Mobilities.

[30] Gimeno-Feliu L.A., Pastor-Sanz M., Poblador-Plou B., Calderón-Larrañaga A., Díaz E., Prados-Torres A. (2021). Overuse or underuse? Use of healthcare services among irregular migrants in a north-eastern Spanish region. Int. J. Equity Health.

[31] Ramsay N., Hossain R., Moore M., Milo M., Brown A. (2019). Health Care While Homeless: Barriers, Facilitators, and the Lived Experiences of Homeless Individuals Accessing Health Care in a Canadian Regional Municipality. Qual. Health Res..

[32] Simich L., Wu F., Nerad S. (2007). Status and health security: An exploratory study of irregular immigrants in Toronto. Can. J. Public Health-Rev. Can. Sante Publique.

[33] Theodore N., Gutelius B., Burnham L. (2018). Workplace Health and Safety Hazards Faced by Informally Employed Domestic Workers in the United States. Workplace Health Saf..

[34] Garabiles M.R., Lao C.K., Xiong Y., Hall B.J. (2019). Exploring comorbidity between anxiety and depression among migrant Filipino domestic workers: A network approach. J. Affect. Disord..

[35] Mendoza N.B., Mordeno I.G., Latkin C.A., Hall B.J. (2017). Evidence of the paradoxical effect of social network support: A study among Filipino domestic workers in China. Psychiatry Res..

[36] Hall B.J., Zhang Y., Li K., Garabiles M.R., Latkin C. (2020). Domestic Workers from the Philippines in China: An Opportunity for Health Promotion within the Belt and Road Initiative. J. Immigr. Minority Health.

[37] Haugen H.Ø., Speelman T. (2022). China’s Rapid Development Has Transformed Its Migration Trends. https://www.migrationpolicy.org/article/china-development-transformed-migration.

[38] Yan M., Chen F. (2009). Reflections on the Investigation and Management of “Filipino Domestic Helpers” in Hangzhou. Public Secur. Sci. J. J. Zhejiang Police Coll..

[39] Yang G., Wang C. (2018). A Study on the Possible Policies in Governing Filipino Domestic Workers in Mainland China. J. Beijing Police Coll..

[40] Wang H., Huang B. (2021). Study of the governance of Foreign Domestic workers in China. J. Beijing Police Coll..

[41] Hurun Research Institute (2020). Demand Management of High Net Worth Individuals in China(2020)imd.

[42] Lin X. (2018). “Purchasing Hope”:The Consumption of Children Education in Urban China. Sociol. Res..

[43] Creswell J.W. (1998). Qualitative Inquiry and Research Design: Choosing among Five Traditions.

[44] Lambert V.A., Lambert C.E. (2012). Qualitative descriptive research: An acceptable design. Pac. Rim Int. J. Nurs. Res..

[45] Colorafi K.J., Evans B. (2016). Qualitative Descriptive Methods in Health Science Research. Herd-Health Environ. Res. Des. J..

[46] Morse J.M. (2010). How Different is Qualitative Health Research from Qualitative Research? Do We Have a Subdiscipline?. Qual. Health Res..

[47] Parker C., Scott S., Geddes A. (2019). Snowball Sampling. SAGE Research Methods Foundations.

[48] Cohen N., Arieli T. (2011). Field research in conflict environments: Methodological challenges and snowball sampling. J. Peace Res..

[49] Sadler G.R., Lee H.-C., Lim R.S.-H., Fullerton J. (2010). Recruitment of hard-to-reach population subgroups via adaptations of the snowball sampling strategy. Nurs. Health Sci..

[50] Bryman A. (2016). Social Research Methods.

[51] Francis J.J., Johnston M., Robertson C., Glidewell L., Entwistle V., Eccles M.P., Grimshaw J.M. (2010). What is an adequate sample size? Operationalising data saturation for theory-based interview studies. Psychol. Health.

[52] Saunders B., Sim J., Kingstone T., Baker S., Waterfield J., Bartlam B., Burroughs H., Jinks C. (2018). Saturation in qualitative research: Exploring its conceptualization and operationalization. Qual. Quant..

[53] López-Domene E., Granero-Molina J., Fernández-Sola C., Hernández-Padilla J.M., López-Rodríguez M.d.M., Fernández-Medina I.M., Guerra-Martín M.D., Jiménez-Lasserrrotte M.d.M. (2019). Emergency Care for Women Irregular Migrants Who Arrive in Spain by Small Boat: A Qualitative Study. Int. J. Environ. Res. Public Health.

[54] Regmi P.R., Teijlingen E.V., Mahato P., Aryal N., Jadhav N., Simkhada P., Zahiruddin Q.S., Gaidhane A. (2019). The Health of Nepali Migrants in India: A Qualitative Study of Lifestyles and Risks. Int. J. Environ. Res. Public Health.

[55] Braun V., Clarke V. (2006). Using thematic analysis in psychology. Qual. Res. Psychol..

[56] Hennink M.M., Kaiser B.N., Marconi V.C. (2017). Code Saturation versus Meaning Saturation: How Many Interviews Are Enough?. Qual. Health Res..

[57] Tong A., Sainsbury P., Craig J. (2007). Consolidated criteria for reporting qualitative research (COREQ): A 32-item checklist for interviews and focus groups. Int. J. Qual. Health Care.

[58] Figueiredo M.d.C., Suleman F., Botelho M.d.C. (2018). Workplace Abuse and Harassment: The Vulnerability of Informal and Migrant Domestic Workers in Portugal. Soc. Policy Soc..

[59] Bakan A.B., Stasiulis D.K. (1997). Not one of the family: Foreign domestic workers in Canada. Contemp. Sociol..

[60] Hall B.J., Pangan C.A.C., Chan E.W., Huang R.L. (2019). The effect of discrimination on depression and anxiety symptoms and the buffering role of social capital among female domestic workers in Macao, China. Psychiatry Res..

[61] Kayano M., Yoshiuchi K., Al-Adawi S., Viernes N., Dorvlo A.S., Kumano H., Kuboki T., Akabayashi A. (2008). Eating attitudes and body dissatisfaction in adolescents: Cross-cultural study. Psychiatry Clin. Neurosci..

[62] Jureidini R., Moukarbel N. (2004). Female Sri Lankan domestic workers in Lebanon: A case of ‘contract slavery’?. J. Ethn. Migr. Stud..

[63] Sporton D. (2013). ‘They Control My Life’: The Role of Local Recruitment Agencies in East European Migration to the UK. Popul. Space Place.

