# Supplementary material for: Irregular Migrant Workers and Health: A Qualitative Study of Health Status and Access to Healthcare of the Filipino Domestic Workers in Mainland China
Source: Healthcare (Basel). 2022 Jun 27;10(7):1204. doi: 10.3390/healthcare10071204 (PMC9324804; doi:10.3390/healthcare10071204)
Supplement: Supplementary file 1 [file healthcare-10-01204-s001.zip › healthcare-1745407-supplementary.pdf]

Table S1. Consolidated criteria for reporting qualitative studies (COREQ): 32-item checklist.

No	Item	Guide questions/description	Response
**Domain 1: Research team and reflexivity**			
Personal Characteristics			
Interviewer/facilitator	1	Which author/s conducted the interview or focus group?	Author A conducted interview.
Credentials	2	What were the researcher's credentials? E.g. PhD, MD	Author A was a PhD candidate, Author B, C and D had a PhD.
Occupation	3	What was their occupation at the time of the study?	Author A was a PhD candidate, Author B was an Assistant Professor, Author C and D were professors.
Gender	4	Was the researcher male or female?	Author A and C were female and Author B and D were male.
Experience and training	5	What experience or training did the researcher have?	All researchers had experience in carrying out qualitative research.
Relationship with participants			
Relationship established	6	Was a relationship established prior to study commencement?	No.
Participant knowledge of the interviewer	7	What did the participants know about the researcher? e.g. personal goals, reasons for doing the research	Name, occupation, purpose for carrying out the research.
Interviewer characteristics	8	What characteristics were reported about the interviewer/facilitator? e.g. Bias, assumptions, reasons and interests in the research topic	Name, occupation, contact method, purpose for carrying out the research.
**Domain 2: Study design**			
Methodological orientation and Theory	9	What methodological orientation was stated to underpin the study? e.g. grounded theory, discourse analysis, ethnography, phenomenology, content analysis	A qualitative descriptive approach, with content and theme analysis.
Participant selection			
Sampling	10	How were participants selected? e.g. purposive, convenience, consecutive, snowball	Snowball sampling.
Method of approach	11	How were participants approached? e.g. face-to-face, telephone, mail, email	Face to face interview.
Non-participation	13	How many people refused to participate or dropped out? Reasons?	None
Setting			
Setting of data collection	14	Where was the data collected? e.g. home, clinic, workplace	Informant's home, boarding house.
Presence of non-participants	15	Was anyone else present besides the participants and researchers?	There were specialists who could offer psychological consideration in Professor D's lab.

Description of sample	16	What are the important characteristics of the sample? e.g. demographic data, date	On average, these Filipinos had been working in mainland China for 7.82 years(some have work experience in other areas). Their average age was 42 years old.
Data collection			
Interview guide	17	Were questions, prompts, guides provided by the authors? Was it pilot tested?	Yes. / Yes.
Repeat interviews	18	Were repeat interviews carried out? If yes, how many?	No.
Audio/visual recording	19	Did the research use audio or visual recording to collect the data?	Audio recording.
Field notes	20	Were field notes made during and/or after the interview or focus group?	After
Duration	21	What was the duration of the interviews or focus group?	Average 53 minutes.
Data saturation	22	Was data saturation discussed?	Yes.
Transcripts returned	23	Were transcripts returned to participants for comment and/or correction?	They were reviewed by 2 key informants.
**Domain 3: analysis and findings**			
Data analysis			
Number of data coders	24	How many data coders coded the data?	Two (Authors A, D)
Description of the coding tree	25	Did authors provide a description of the coding tree?	Yes.
Derivation of themes	26	Were themes identified in advance or derived from the data?	Themes were derived by using both methods.
Software	27	What software, if applicable, was used to manage the data?	Nvivo 12 plus.
Participant checking	28	Did participants provide feedback on the findings?	Yes./They were reviewed by 2 key informants.
Reporting			
Quotations presented	29	Were participant quotations presented to illustrate the themes/findings? Was each quotation identified? e.g. participant number	Yes. / Yes.
Data and findings consistent	30	Was there consistency between the data presented and the findings?	Yes.
Clarity of major themes	31	Were major themes clearly presented in the findings?	Yes.
Clarity of minor themes	32	Is there a description of diverse cases or discussion of minor themes?	Yes.
